# Computational analysis and modeling of cleavage by the immunoproteasome and the constitutive proteasome

**DOI:** 10.1186/1471-2105-11-479

**Published:** 2010-09-23

**Authors:** Carmen M Diez-Rivero, Esther M Lafuente, Pedro A Reche

**Affiliations:** 1 Laboratory of Immunomedicine, Department of Microbiology I-Immunology, Facultad de Medicina, Universidad Complutense de Madrid, Ave Complutense S/N, Madrid 28040, Spain; 2 Department of Microbiology I-Immunology, Facultad de Medicina, Universidad Complutense de Madrid, Ave Complutense S/N, Madrid 28040, Spain

## Abstract

**Background:**

Proteasomes play a central role in the major histocompatibility class I (MHCI) antigen processing pathway. They conduct the proteolytic degradation of proteins in the cytosol, generating the C-terminus of CD8 T cell epitopes and MHCI-peptide ligands (*P1 *residue of cleavage site). There are two types of proteasomes, the constitutive form, expressed in most cell types, and the immunoproteasome, which is constitutively expressed in mature dendritic cells. Protective CD8 T cell epitopes are likely generated by the immunoproteasome and the constitutive proteasome, and here we have modeled and analyzed the cleavage by these two proteases.

**Results:**

We have modeled the immunoproteasome and proteasome cleavage sites upon two non-overlapping sets of peptides consisting of 553 CD8 T cell epitopes, naturally processed and restricted by human MHCI molecules, and 382 peptides eluted from human MHCI molecules, respectively, using *N-grams*. Cleavage models were generated considering different epitope and MHCI-eluted fragment lengths and the same number of C-terminal flanking residues. Models were evaluated in 5-fold cross-validation. Judging by the Mathew's Correlation Coefficient (*MCC*), optimal cleavage models for the proteasome (*MCC *= 0.43 ± 0.07) and the immunoproteasome (*MCC *= 0.36 ± 0.06) were obtained from 12-residue peptide fragments. Using an independent dataset consisting of 137 HIV1-specific CD8 T cell epitopes, the immunoproteasome and proteasome cleavage models achieved *MCC *values of 0.30 and 0.18, respectively, comparatively better than those achieved by related methods. Using ROC analyses, we have also shown that, combined with MHCI-peptide binding predictions, cleavage predictions by the immunoproteasome and proteasome models significantly increase the discovery rate of CD8 T cell epitopes restricted by different MHCI molecules, including A*0201, A*0301, A*2402, B*0702, B*2705.

**Conclusions:**

We have developed models that are specific to predict cleavage by the proteasome and the immunoproteasome. These models ought to be instrumental to identify protective CD8 T cell epitopes and are readily available for free public use at http://imed.med.ucm.es/Tools/PCPS/.

## Background

CD8 cytotoxic T cells play a key role fighting intracellular pathogens, eliminating infected cells that display on their cell surface foreign peptides bound to major histocompatibility complex class I (MHCI) molecules [1-3]. CD8 T cell epitopes and, in general, peptides presented by MHCI molecules, derive from protein fragments produced in the cytosol by the proteolytic action of the proteasome [4,5]. Briefly, the proteasome generates protein fragments between 7 and 15 amino acids. Some of these peptides can be transported from the cytosol into the endoplasmic reticulum (ER) by the transporter associated with antigen processing (TAP), where they can be loaded onto nascent MHCI molecules. Interestingly, whereas different peptidases and proteases in the cytosol and the endoplasmic reticulum shape the N-terminus of the peptides presented by MHCI molecules [6], their C-terminus generally corresponds to the *P1 *residue of the proteasome cleavage site [7,8].

The proteasome is a multisubunit ATP-dependent protease and it is primarily responsible for the degradation of cytosolic proteins [9]. The most common form of the proteasome is known as the 26 S proteasome, which is composed by a catalytic core (20S) and two regulatory complexes (19S), located one at each side of the core [5]. The catalytic activity of the proteasome is located at the subunits β5 (X, LMP7), β2 (Z, MECL-1) and β1 (Y, LMP2) of the 20 S core, which cut after the C-terminus of hydrophobic (chymotrypsin-like activity), basic (trypsin-like activity) or acidic (caspase-like activity) amino acids, respectively [10]. Upon IFN-γ exposure, the three catalytic subunits of the constitutive 20 S core can be replaced by three new catalytic subunits: β5i (LMP2), β2i (MECL-1), and β1i (LMP2) [11]. This new form of proteasome is called immunoproteasome, as opposed to the constitutively expressed proteasome. The immunoproteasome is the constitutive form of proteasome presented in dendritic cells [12]. The immunoproteasome produces different but overlapping cleavage patterns with regard to those of the proteasome [13]; chiefly, the immunoproteasome does not cut after acidic residues [13,14]. Because the antigen-specific cytotoxic function of CD8 T cells is generally acquired upon the recognition of MHCI-bound peptide antigens displayed on the cell surface of dendritic cells (priming), it is likely that protective epitopes are those generated by the proteasome and the immunoproteasome [15].

Prediction of proteasome cleavage sites is relevant for CD8 T cell epitope identification and, subsequently, for the design of epitope-based vaccines eliciting CD8 T cell responses. Therefore, different methods to predict proteasome cleavage sites have been reported. Proteasome cleavage prediction methods were first developed using enolase and β-casein protein fragments generated *in vitro *by human constitutive proteasomes [16-18]. Likewise, a kinetic model of the proteasome proteolytic activity was also developed using peptide fragments from *in vitro *digestions [19,20]. Those models are specific for the constitutive 20 S proteasome that was used to generate the peptide fragments. Proteasome cleavages take place between the C-terminus of MHCI-restricted peptides (*P1 *residue of cleavage site) and their most proximal C-terminal flanking residue (*P1' *residue of cleavage site). Therefore, proteasome cleavage prediction methods have also been developed using MHCI-restricted peptide ligands and their C-terminal flanking regions [21-23]. These latter methods appear to outcompete the former methods that were trained on actual proteolytic digestion data on the task of predicting cleavage sites defined by MHC I restricted peptides [24]. However, methods trained on experimental cleavage data can be more suitable for identifying protein fragments produced by the proteasome [18].

The problem of predicting proteasome cleavage sites resembles that of modeling grammatical rules. Therefore, in this manuscript, we have applied statistical language models [25] to analyze and model the cleavage sites of the constitutive proteasome and the immunoproteasome. Proteasome cleavage sites were obtained from MHCI-eluted peptides and their C-terminal flanking regions, whereas immunoproteasome cleavage sites were rendered from naturally processed CD8 T cell epitopes and their C-terminal flanking regions. In cross-validation, optimal proteasome and immunoproteasome cleavage models achieved an *MCC *of 0.43 ± 0.07 and 0.36 ± 0.06, respectively. These models were trained using 12-residue fragments, consisting of the C-terminal end of MHCI-restricted peptides (*P6 *- *P1 *residues of cleavage site) followed by the 6 most-proximal C-terminal flanking residues (*P1' *- *P6' *residues of cleavage site). The fact that optimal models were trained using peptide fragments consisting of 6 amino acids at each side of the cleavage site is consistent with the activity exhibited by the proteasome [26]. Here, we have also shown that combining cleavage predictions by the constitutive and the immunoproteasome with MHCI-binding predictions serve to improve the prediction rate of CD8 T cell epitopes. Cleavage predictions using our models are available at http://imed.med.ucm.es/Tools/PCPS/.

## Methods

### Datasets and sequences

We assembled three non-overlapping datasets consisting of distinct MHCI-restricted peptides and their protein sources. The peptide content in these datasets was as follows. The first dataset encompassed 553 CD8 T cell epitopes from different sources but from Human Immunodeficiency Virus (HIV1) and were all restricted by human MHCI molecules. Immune responses against these epitopes have been verified experimentally using T cells from infected humans. Because CD8 T cell immune responses against these epitopes are elicited in the course of an infection, we assume that they are naturally processed. The second dataset included 382 peptides that were eluted from human MHCI molecules, and the third dataset encompassed 137 HIV1-specific CD8 T cell epitopes restricted by human MHCI molecules and naturally processed. MHCI-restricted peptides in these datasets were collected from the EPIMHC [27], Immuneepitope [28] and Los Alamos databases [29], and consisted of unique nonapeptides (9-mers) that were subjected to a sequence similarity reduction schema using the *purge *utility implemented in the Gibbs Sampler [30]. As a result, peptides in these three datasets do not share more than 4 identical residues (global sequence similarity in the first, second and third datasets is 3.1 ± 11.7, 3.9 ± 12.8, and 3.5 ± 11.7, respectively). Moreover, in all datasets the same MHCI molecule restricts less than 18% of all peptides. In additional file 1, we show the distribution of commonly expressed MHCI alleles in each of the three datasets. The corresponding author will also provide these datasets upon written request.

### Model building and evaluation

Cleavage models were trained and evaluated on datasets consisting of peptide fragments of the same length derived from MHCI-eluted peptides (proteasome models) and CD8 T cell epitopes (immunoproteasome models) and their C-terminal flanking regions, using the NGRAM-COUNT utility implemented by the SRILM package [25]. Peptide fragments encompassed two portions with the same number of residues, one fraction consisting of the C-terminal end of MHCI-eluted peptides or CD8 T cell epitopes, and the other one of their C-terminal flanking region. Cleavage sites -defined between the C-terminus of MHCI-restricted peptides (*P1 *residue of cleavage site) and the most proximal C-terminal flanking residue (*P1' *residue)- were indicated by a "|" symbol. Cleavage models were generated considering peptide fragments ranging from 4 to 18 residues. Representative peptide fragments of 6 and 12 amino acids are C T L | T I G and P S C C T L | T I G V S S, respectively, where C T L and P S C C T L are two C-terminal portions of the peptide and T I G and T I G V S S are C-terminal flanking residues drawn from the protein source. Cleavage models were tested and evaluated at different thresholds using the SRLIM HIDDEN-NGRAM utility. HIDDEN-NGRAM is a word boundary program that uses *N-gram *models [25] produced by NGRAM-COUNT to predict the probability of hidden tags -cleavage sites- in any peptide fragment. The evaluation of the models was carried out through 5-fold cross-validation experiments that were repeated 5 times, obtaining mean estimations and standard deviations of the measures of performance indicated below.

### Measures of performance

Cleavage predictions were examined in each residue at different probability thresholds (*th) *and were judged following the schema proposed in previous works [22,24]. It is assumed that cleavage sites should preferentially occur after the C-terminus of MHCI-restricted peptides (*P1 *residue of cleavage site) than over any other position within the peptide. Under such schema, any given test peptide was classified as follows:

- TP (True positive): Cleavage score at the C-terminus (*P1 *residue of cleavage site) is above the *th*.

- FN (False negative): Cleavage score of *P1 *residue is below the *th*.

- TN (True negative): All the residues within the test fragment have a cleavage score bellow the *th*. Alternatively, if there are residues with cleavage scores above the *th*, but smaller than that of the *P1 *residue.

- FP (False positive): There is at least one residue within the peptide with a cleavage score that is both, above the *th *and above that of the *P1 *residue.

Upon this classification approach, we computed the Sensitivity (*SE*), Specificity (*SP*) and Matthews correlation coefficient (*MCC*) [31] of the predictions using Equations 1, 2 and 3, respectively,

(1) $$SE=\frac{TP}{TP+FN}$$

(2) $$SP=\frac{TN}{TN+FP}$$

(3) $$MCC=\frac{\left(TP*TN\right)-\left(FN*FP\right)}{\sqrt{\left(TN+FN\right)\left(TP+FN\right)\left(TN+FP\right)\left(TP+FP\right)}}$$

In addition, we also computed the parameter *BTR *(Better Than Random) which was first introduced by Reche *et al. *[32] to compare the *SE *of a given model and that of a random model producing the same number of cleavage sites (Equation 4).

(4) BTR=SE−ECS

*ECS *(Expected Cleavage Sites) represents the ratio of cleavage sites correctly predicted by a model that distributes cleavage sites randomly and is given by Equation 5.

(5) $$ECS=\frac{C}{F*N}$$

Where *C *is the total number of cleavage sites (above the *th*) predicted by a given cleavage model in a test set of peptide fragments -specifically, within the MHCI-restricted peptide portion of the peptide fragment-; *F *is the number of MHCI-restricted peptide residues included in the peptide fragments used for training and testing; and *N *is the total number of peptide fragments in the dataset. Note that peptide fragments used for model building and evaluation encompassed two portions with the same number of residues, one consisting of the C-terminal end of MHCI-restricted peptides and the other of their C-terminal flanking region (details elsewhere in Methods). *ECS *is somewhat equivalent to the *SE *of a model that distributes all the cleavage sites randomly. Thus, the bigger the difference between *SE *and *ECS *the better the predictions produced by the model.

### Prediction of peptide binding to MHCI

We used Position Specific Scoring Matrices (PSSMs) to compute binding scores of peptides to the relevant MHCI molecules [33]. Actual binding of peptides to a particular MHCI molecule was assessed relating its binding score to those of 10000 reference peptides, 9-mers randomly obtained from SwissProt, computed using the same relevant PSSM. Thus, a given peptide was considered to bind a specific MHCI molecule when its binding score ranked among the *X *percentile (threshold) of top binding scores. The same peptide was considered not to bind to that MHCI if it ranked below the *X *percentile of top binding scores. PSSMs are derived from alignments of peptides of the same size known to bind to a given MHCI molecule [32,34,35]. Given that MHCI-bound peptides are usually of 9 residues of length, in this study we used PSSMs specific for the prediction of peptide binders of that length (9mers).

### ROC analysis

We used 5 different sets of CD8 T cell epitopes consisting of 316, 50, 70, 47 and 30 peptides restricted by A*0201, A*0301, A*2402, B*0702, and B*2705, respectively, to evaluate the discovery rate of CD8 T cell epitopes using MHCI peptide-binding predictions alone, or in combination with proteasome cleavage predictions. Receiver operating characteristic (*ROC*) curves [36] were used to analyze the predictions. In the ROC analysis, we represented the *SE *(Equation 1) *versus 1-SP *(Equation 2) of the T cell epitope predictions obtained over a continuous range of percentile thresholds of MHCI binding (detail elsewhere in Methods). Non-T cell epitopes, required to compute the *SP *of the predictions, consisted of peptides of 9 residues randomly selected from the SwissProt database. A 1:3 ratio of T cell epitopes to non-T cell epitopes data was used. When evaluating the combination of MHCI binding and proteasome cleavage predictions, we applied a filtering approach such as that used by Dönnes and Kohlbacher [37]. Under this approach, peptides that are not predicted to be cleaved by the proteasome are discarded prior to the ROC analysis.

The area under ROC curves (*AUC*) was used as a global threshold-independent measure of performance. The maximum accuracy corresponds to an *AUC *= 1 while an *AUC *= 0.5 is indicative of a random prediction. Predictions are poor for values of *AUC *> 0.7, good for values of *AUC *> 0.8 and excellent for values of *AUC *> 0.9. ROC analyses were repeated 10 times, using the same T cell epitopes but different non-T cell epitopes. Thus, we obtained confident values of *AUC *(mean and standard deviation). Statistical significance of the differences between *AUC *values was evaluated using standard one-side two sample Student *t- *tests (*p *< = 0.05).

### Web implementation

Immunoproteasome and proteasome cleavage models were implemented for free public use on the Web using a PERL CGI (Common Gateway Interface) script that executes the predictions on user-provided input data and returns the results to the browser. In addition, we used JavaScript for handling and verification of the input data before submission. Proteasome and immunoproteasome cleavage models exhibited optimal predictions at different model-specific cleavage scores. Therefore, cleavage scores by the different models were normalized and standardized so that cleavage sites are predicted at a score ≥ 0.5.

## Results

### Proteasome and immunoproteasome cleavage models

Cleavage models were generated from two types of MHCI-restricted peptides and their flanking regions using *N-grams*. *N-gram *models are frequently applied to speech recognition and natural language tagging [38], but they have also been applied to sequence analysis and motif identification [32,39-41]. We built two types of cleavage models. Immunoproteasome cleavage models were built upon a dataset encompassing 553 CD8 T cell epitopes that have been reported to be recognized by humans during the course of an infection. Epitope-specific CD8 T cell responses are generally primed by dendritic cells which express the immunoproteasome. Therefore, naturally processed CD8 T cell epitopes can be used to reproduced the cleavage by the immunoproteasome. In contrast, proteasome cleavage models were based on a set of 382 peptides that were eluted from human MHCI molecules. Peptide elution experiments are generally carried out using various types of cells (virtually never dendritic cells) and under conditions that do not induce the expression of the immunoproteasome. Therefore, we considered that MHCI-eluted peptides are produced by the proteasome. A detailed description of these datasets is elsewhere in Methods.

**Figure 1:**
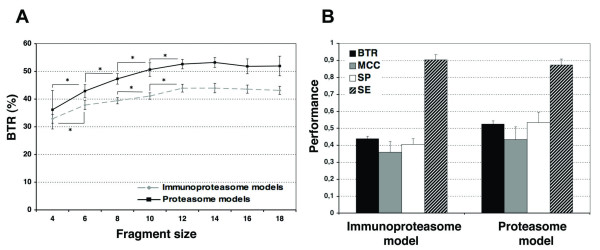
**Evaluation of immunoproteasome and proteasome prediction models**. The predictive performance of proteasome models was evaluated in 5 fold cross-validation experiments using *MCC*, *BTR*, *SP *and *SE *as measures of performance. Proteasome models were built and tested using MHCI-eluted peptide ligands whereas immunoproteasome models were built and tested using MHCI-restricted CD8 T cell epitopes. A) Predictive performance (*BTR*) achieved by immunoproteasome (*grey line*) and proteasome (*black line*) models trained and tested on peptides of different fragment lengths (abscissa). Statistically significant increments in *BTR *are indicated with "*" symbols. B) Predictive performance (*BTR*, *MCC*, *SP*, *SE*) achieved by the selected proteasome and immunoproteasome models built on peptide fragments of 12 residues.

Numerous immunoproteasome and proteasome cleavage models were obtained from different training sets consisting of peptide fragments varying from 4 to 18 residues -in a given training set, all the peptides have the same size. Peptide fragments used for training included the C-terminus (*P1 *residue of cleavage site) of MHCI-restricted peptides (CD8 T cell epitopes and MHCI-eluted peptides) and comprised two distinct portions with the same number of residues: one consisting of the C-terminal end of MHCI-restricted peptides and the other one of their C-terminal flanking region (see Methods section for more details). Cleavage models were evaluated in 5-fold cross-validation experiments, considering a continuous range of cleavage thresholds. As measures of performance we computed *SE*, *SP*, *MCC *and *BTR *(see Methods section for details), but trusted *BTR *as the key measure of the goodness of the predictions. In Figure 1A we show the optimal *BTR *achieved by the cleavage models with regard to the size of the peptide fragments used for training. A complete summary of the performance of the cleavage models, which also includes the *MCC*, *SE*, *SP *of the predictions, is shown in Table 1.

**Table 1:** Predictive performance of immunoproteasome and proteasome cleavage models

Immunoproteasome					
**Size**	***SE***	***SP***	***ECS***	***MCC***	***BTR***

4	0.807 ± 0.030	0.851 ± 0.039	47.828 ± 2.001	0.660 ± 0.038	0.329 ± 0.016
6	0.763 ± 0.036	0.708 ± 0.042	38.495 ± 0.614	0.472 ± 0.069	0.378 ± 0.016
8	0.906 ± 0.023	0.545 ± 0.038	51.219 ± 1.008	0.484 ± 0.059	0.394 ± 0.011
10	0.802 ± 0.024	0.462 ± 0.019	39.083 ± 1.003	0.281 ± 0.045	0.411 ± 0.012
12	0.903 ± 0.031	0.407 ± 0.031	46.339 ± 0.481	0.357 ± 0.062	0.439 ± 0.014
14	0.872 ± 0.035	0.374 ± 0.023	43.190 ± 1.498	0.284 ± 0.056	0.434 ± 0.017
16	0.855 ± 0.030	0.306 ± 0.041	41.908 ± 1.406	0.193 ± 0.047	0.436 ± 0.015
18	0.857 ± 0.031	0.290 ± 0.028	42.536 ± 1.081	0.179 ± 0.039	0.432 ± 0.015

**Proteasome**					

**Size**	***SE***	***SP***	***ECS***	***MCC***	***BTR***

4	0.803 ± 0.125	0.871 ± 0.052	44.110 ± 9.249	0.681 ± 0.089	0.362 ± 0.069
6	0.792 ± 0.048	0.723 ± 0.037	36.274 ± 1.943	0.516 ± 0.082	0.429 ± 0.023
8	0.855 ± 0.037	0.603 ± 0.047	38.160 ± 2.112	0.473 ± 0.072	0.473 ± 0.019
10	0.885 ± 0.050	0.537 ± 0.046	37.839 ± 2.355	0.452 ± 0.069	0.506 ± 0.025
12	0.874 ± 0.034	0.534 ± 0.062	34.970 ± 1.704	0.434 ± 0.075	0.526 ± 0.017
14	0.871 ± 0.037	0.468 ± 0.065	33.699 ± 1.432	0.371 ± 0.085	0.532 ± 0.018
16	0.844 ± 0.058	0.403 ± 0.065	32.657 ± 1.692	0.276 ± 0.096	0.518 ± 0.027
18	0.794 ± 0.077	0.392 ± 0.060	27.510 ± 1.978	0.206 ± 0.126	0.519 ± 0.035

Cleavage models were built on peptide fragments of a given size encompassing the C-terminal end of MHCI-eluted ligands (proteasome model) or CD8 T cell epitopes (immunoproteasome) and the corresponding C-terminal flanking residues. Predictive performance was evaluated in 5-fold cross-validation experiments. *SE*: sensitivity; *SP*: specificity; *MCC*: Matthew's correlation coefficient; *BTR*: better than random (Eq. 4)

The predictive performance of the cleavage models significantly increased (*p *< 0.05) with the length of the peptide fragments used for training, picking at a fragment size of 12-14 residues (Figure 1A); *BTR *= 0.44 ± 0.02 for the immunoproteasome model and *BTR *= 0.53 ± 0.02 for the proteasome model. In general, the predictive performance of proteasome cleavage models built upon MHCI-eluted peptides was higher than that achieved by immunoproteasome cleavage models, regardless of the length the peptides fragments used for training (Figure 1A). Increasing the size of the peptide fragments beyond 14 residues did not improve the predictive performance of the cleavage models (Figure 1A). Judging the predictions by the *MCC*, the immunoproteasome and proteasome models that were built on peptide fragments of 12 residues (Table 1) achieved the best results. Because no statistical difference was observed between the *BTR *achieved by the models trained on 12 and 14 residues, for further analysis, we used the models trained on 12-residue peptide fragments. The performance of the selected proteasome and immunoproteasome models is summarized in Figure 1B.

### Comparison of the immunoproteasome and proteasome cleavage models

**Figure 2:**
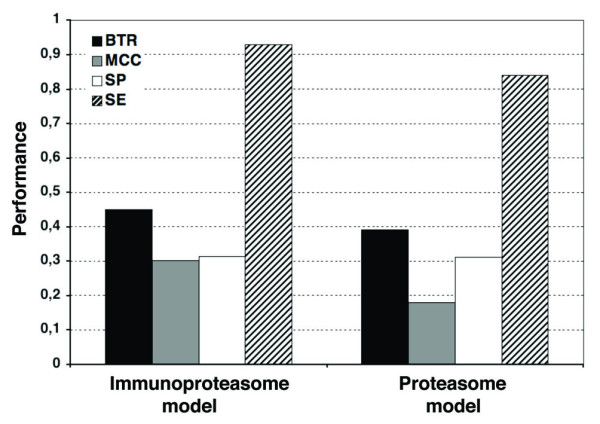
**Model evaluation using an independent test dataset**. The proteasome and immunoproteasome models were evaluated using an independent test consisting of HIV1-specific CD8 T cell epitopes. The predictive performance was evaluated using *BTR *(*black bars*), *MCC *(*grey bars*), *SP *(*white bars*) and *SE *(*pattern bars*).

For further comparisons, we evaluated the immunoproteasome and proteasome cleavage models in an independent test set built from 137 HIV1-specific CD8 T cell epitopes and their flanking regions (Figure 2). The immunoproteasome model achieved better results than the proteasome model, as judged by both, the *BTR *(0.45 for the immunoproteasome model and 0.39 for the proteasome model) and the *MCC *(0.30 for the immunoproteasome model and 0.18 for the proteasome model). These results indicate that the immunoproteasome model appears to be more suitable than the proteasome model to predict the cleavage sites defined by CD8 T cell epitopes.

**Figure 3:**
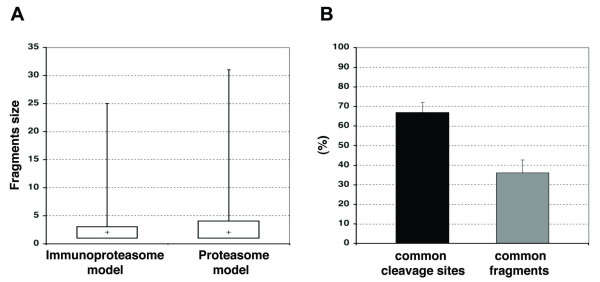
**Analysis of fragmentation patterns produced by the immunoproteasome and proteasome models**. A) BoxPlot of the fragment size distribution obtained with the immunoproteasome and the proteasome models. Not significant difference was observed, using a Wilcoxon test, between the sizes of the fragments generated by each model B) Overlap between the fragments generated by the immunoproteasome and proteasome models represented as the percentage of common sites (*black bar*) and the percentage of common fragments (*grey bar*). Fragments were obtained from 100 proteins randomly selected from the Swissprot database. We used the optimal proteasome and immunoproteasome models, which were built on 12-residue peptide fragments.

Using the immunoproteasome and proteasome cleavage models, we analyzed the fragmentation patterns resulted from 100 proteins randomly selected from the SwissProt database (Figure 3). The immunoproteasome cleavage model generated fragments with a mean size of 2.23 ± 1.61 residues, whereas the proteasome cleavage model generated fragments with a mean size of 3.02 ± 2.33 residues. Using a Wilcoxon test, we observed no significant difference between the sizes of the fragments generated with the proteasome and immunoproteasome models (Figure 3A). This analysis also revealed that 36% of the peptide fragments generated by the proteasome and immunoproteasome are identical, and 67% of the cleavage sites are shared (Figure 3B).

### Comparison with NetChop

**Figure 4:**
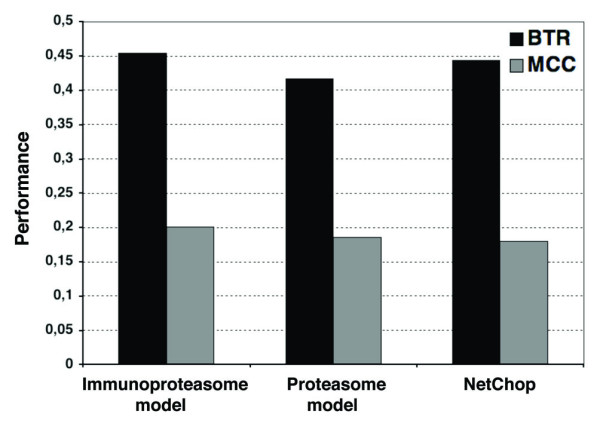
**Comparative analysis of cleavage predictions**. The figure depicts the *MCC *(*black bars*) and the *BTR *(*grey bars*) achieved by our immunoproteasome and proteasome models and NetChop on an independent test set of 137 HIV1-specific CD8 T cell epitopes. Because NetChop was built using complete nonameric MHCI-restricted peptides, in this analysis we have evaluated the cleavage predictions by the three models over the entire length of the T cell epitopes being tested.

We also used the 137 HIV1-specific CD8 T cell epitopes and their flanking regions to compare the cleavage predictions obtained with our *N-gram *cleavage models and those obtained using the NetChop web sever. The NetChop system uses an artificial neural-network model that was built upon MHCI-restricted peptides [21]. For this comparison, we used NetChop default settings (cleavage sites occur after residues having a probability of 0.5 or higher) in computing the *SE*, *SP*, and *MCC *of the predictions following the same schema reported by the NetChop developers [22] (see Methods section for details). In addition, we computed the *BTR *parameter defined in this study. Because NetChop models were trained on 18-residue peptide fragments consisting of full-length MHCI-restricted peptides (9 residues) and the most proximal 9 residues flanking the C-terminus, in this comparison we evaluated *SE*, *SP*, *MCC *and *BTR *on peptide fragments consisting of the full-length HIV1-specific CD8 T cell epitopes. Note that in previous analyses these parameters were evaluated on the portion of the peptide fragments corresponding to the MHCI-restricted peptides. The results of this analysis are depicted in Figure 4. The immunoproteasome and proteasome *N-gram *models achieved *MCC *values (0.20 and 0.19, respectively) similar to those obtained using NetChop (0.18). Likewise, NetChop and our *N-gram *models achieved similar *BTR *values around 0.44 (Figure 4).

### Combination of MHCI-peptide binding and cleavage predictions

**Figure 5:**
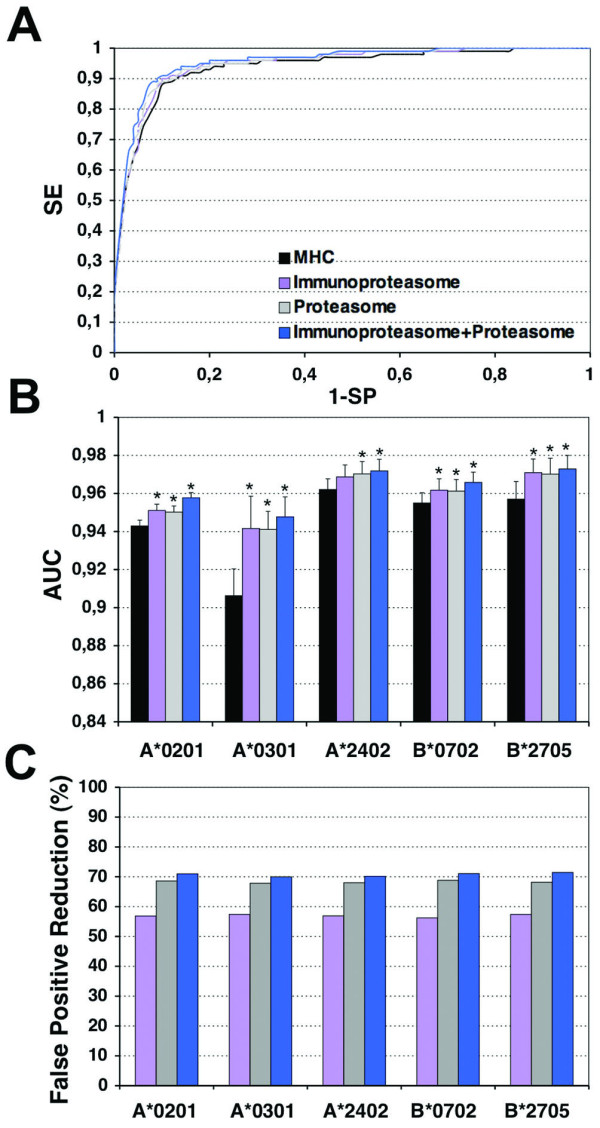
**Prediction of T cell epitopes using MHCI-peptide binding and cleavage models**. A) ROC curves depicting the prediction of T cell epitopes restricted by A*0201 using MHCI-peptide binding prediction alone (*black line*) or in combination with cleavage predictions by the immunoproteasome (*purple line*), the proteasome (*grey line*) and both cleavage models together (*blue line*). B) *AUC *values obtained for the prediction of T cell epitopes restricted by 5 different HLA I molecules (A*0201, A*0301, A*2402, B*0702, B*2705) using MHCI-peptide binding predictions alone (*black bars*) and in combination with cleavage predictions by the immunoproteasome (*purple bars*), proteasome (*grey bars*) and both cleavage models together (*blue bars*). Significant increases (*p *< 0.05) in *AUC *values with regard to MHCI-peptide binding predictions alone (*black bars*) are indicated with the symbol "*". C) Reduction of false positives. The figure represents the decrease in percentage of false positives after introducing cleavage predictions by the immunoproteasome (*purple bars*), the proteasome (*grey bars*) and both together (*blue bars*). False positive reduction was computed over the entire ROC analysis.

We also evaluated the impact of combining cleavage and MHCI-peptide binding predictions on T cell epitope identification. Specifically, using a ROC analysis (see Methods section for details), we analyzed the result of such combination to discriminate CD8 T cell epitopes restricted by 5 different MHCI molecules (A*0201, A*0301, A*2402, B*0702 and B*2705) from random peptides. We combined MHCI-peptide binding predictions with cleavage predictions by the immunoproteasome and proteasome models, individually or together, and used *AUC *values (computed after the ROC analyses, see Methods for details) as a measure of the goodness of the predictions (Figure 5).

MHCI-peptide binding predictions alone achieved high *AUC *values above 0.9 -regardless of the MHCI molecule-, that did not leave much margin to observe any large improvements on CD8 T cell epitope predictions. Nevertheless, combining the proteasome and immunoproteasome models separately or together with MHCI-peptide binding predictions resulted in increased *AUC *values (Figure 5B). Moreover, such increases were statistically significant (*p *< 0.05) in all cases. The major increment in *AUC *was observed for A*0301-restricted epitopes. Alone, MHCI-peptide binding predictions reached an *AUC *= 0.9063 ± 0.0141 for A*0301, whereas in combination with the immunoproteasome and proteasome cleavage predictions achieved *AUC *values of 0.9416 ± 0.017, and 0.9411 ± 0.0095, respectively.

There were no differences between the results obtained combining MHCI-peptide binding and the cleavage predictions by the immunoproteasome model or the proteasome model, but the joint combination of both cleavage models (immunoproteasome and proteasome) with the MHCI-peptide binding resulted in *AUC *values larger than those obtained using single cleavage models (Figure 5B). Nevertheless, with the exception of A*0201 (*p *< 0.05), these increases in *AUC *were not statistically significant with regard to those *AUC *obtained using solely either cleavage model (Figure 5B).

Enhanced *AUC *values obtained upon combining the cleavage models with MHCI-peptide binding predictions are due to the reduction of the number of false positives detected with regard to the MHCI-peptide binding predictions alone (Figure 5C). Taking MHCI-peptide binding predictions alone as reference, we observed a ~56% decrease of false positives (computed over the entire range of thresholds used in the *ROC *analysis) when using the immunoproteasome model. The reduction of false positives was even larger (68%) when using the proteasome model and increased slightly when both models were combined (70%).

### Proteasome Cleavage Prediction Server (PCPS)

**Figure 6:**
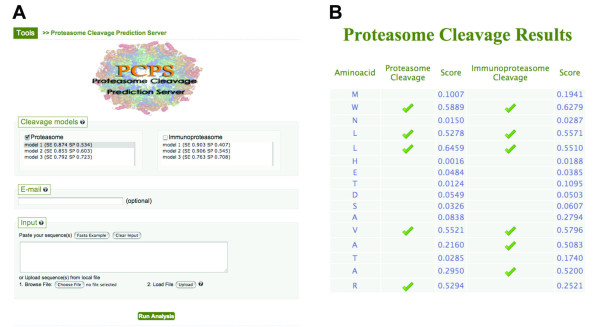
**Proteasome Cleavage Prediction Server (PCPS)**. A) PCPS interface. B) PCPS result page. The page returns the predicted cleavage score after each residue of the protein query. The residues with a cleavage score above the threshold (0.5) are marked with a tick. These residues correspond to the *P1 *residue of the cleavage site and determine the C-terminus of MHCI-restricted peptides.

We developed PCPS (Proteasome Cleavage Prediction Server) to allow the prediction of proteasome and immunoproteasome cleavage through our *N-gram *models. PCPS is available for free public use at http://imed.med.ucm.es/Tools/PCPS/. PCPS was designed to be intuitive and user friendly (Figure 6A). The main input data for PCPS is one or several protein sequences that can be pasted or uploaded to the server in multiple formats, including FASTA, IG, GenBank, EMBL, Phylip, NBRF, GCG, DNAStrider, PIR, MSF, ASN and PAUP. The sequences provided to the server are subjected to a cleavage analysis using *N-gram *models that are selected by the user from the CLEAVAGE MODELS section. There are several models available for both proteasomes, constitutive and immunoproteasome, which differ in sensitivity and specificity, and users can combine different proteasome and immunoproteasome models. Cleavage models in PCPS were trained on peptide fragments of 12 (*models 1*), 8 (*models 2*) and 6 (*models 3*) residues. The models trained on 12 residues exhibited the best performance (*MCC *= 0.43 ± 0.07 for the proteasome cleavage model and *MCC *= 0.36 ± 0.06 for the immunoproteasome cleavage model) (Table 1). The output of PCPS consists of a table indicating the cleavage score of each residue in the protein queries (Figure 6B). Computed scores reflect the likelihood that the proteasome/immunoproteasome would cleave the protein after such residue (*P1 *residue of cleavage site). Whenever the cleavage score is higher than 0.5, a tick marks the corresponding residue. The different models actually differ in the sensitivity, specificity, and BTR of the predictions. In PCPS, the indicated specificity and sensitivity of the models were achieved at cleavage thresholds of 0.5, but users can experiment with the server and decide different cleavage thresholds.

## Discussion

It is generally believed that the C-terminus of most CD8 T cell epitopes, and in general that of most MHCI-restricted peptides, results from the proteolytic cleavage by the proteasome [4,9]. Some other proteases, chiefly tripeptidyl peptidase II (TPP II), also play some role generating the C-terminus of some MHCI-restricted peptides[42-44], specifically through the degradation of some proteolytic products generated by the proteasome that are longer than 15 residues [43]. However, because the majority of the peptide fragments generated by the proteasome are shorter than 15 residues [13], the proteasome is still the principal source of the C-terminus of peptides that are bound to MHCI molecules. As a result, proteasome cleavage models can be derived using cleavage sites recreated from MHCI-restricted peptides and their C-terminal flanking regions [21-23].

There are two types of proteasomes, the immunoproteasome and the constitutive proteasome, which differ in their cleavage patterns [14]. The constitutive proteasome is the form expressed in most nucleated cells, whereas the immunoproteasome is constitutively expressed in mature dendritic cells. Antigen presentation by dendritic cells is generally required to prime and instruct naïve CD8 T cells in an antigen specific manner. Subsequently, the effector function of CD8 T cells is executed upon recognizing the same antigenic peptides on target cells [45]. Consequently, the immunoproteasome is responsible for the generation of the C-terminus of the peptides that elicit the CD8 T cell response, whereas the constitutive proteasome determines the C-terminus of the MHCI-peptide ligands that can be the targets of such response. Protective CD8 T cell epitopes are likely those generated by both, the constitutive proteasome and the immunoproteasome [15].

In this work, we have assumed that MHCI-eluted peptides reflect protein degradation by the proteasome, whereas bona fide identified CD8 T cell epitopes elicited in patients during the course of an infection reflect protein degradation by the immunoproteasome, but not necessarily by the proteasome. The latter is due to the fact that epitope verification is generally carried out by measuring the response of T cells to synthetic peptides loaded onto antigen presenting cells, which are seldom dendritic, thus bypassing antigen processing by the proteasome in the test target cells. Subsequently, using *N-grams*, we have modeled the proteasome and immunoproteasome cleavage from datasets of peptide fragments of different length built upon MHCI-eluted peptides (proteasome model) and CD8 T cell epitopes (immunoproteasome model) and their C-terminal flanking regions. These models predict whether the C-terminus of a given peptide, in the context of its flaking residues, is likely to result from the proteolytic activity of the proteasome and/or the immunoproteasome (*P1 *residue of cleavage site).

The best cleavage predictions for both proteasomes, constitutive and immunoproteasome, were obtained using *N-grams *trained on 12-residue peptide fragments, encompassing the 6 most proximal flanking residues to the C-terminus of the MHCI-restricted peptides preceded by 6 residues from the C-terminal end of the MHCI-restricted peptides (Figure 1). These results are consistent with reports indicating that proteasomes and immunoproteasomes scrutinize between 10 and 12 residues [10,26]. In contrast, related methods for the prediction of proteasome cleavage that are based on MHCI-restricted peptides have been trained using 18 to 20 residue peptide fragments [19,21,23], which, makes these models, regardless of the results, somewhat artificial.

In cross-validation, the predictive performance of proteasome models exceeded that of immunoproteasome models; the best proteasome cleavage model achieved a *BTR *= 0.53 ± 0.02 and an *MCC *= 0.43 ± 0.07, whereas the best immunoproteasome model achieved a *BTR *= 0.44 ± 0.01 and an *MCC *= 0.36 ± 0.06. Despite that both sets of peptides were subjected to the same sequence reduction procedure (See Methods), these results likely reflect that the set of CD8 T cell epitopes is more numerous and arguably more diverse than the set of MHCI-eluted peptide (see Results). Dendritic cells exhibit non-classical pathways on antigen presentation and some can be immunoproteasome independent [45,46], which could actually account for a higher diversity in the epitope dataset. Nonetheless, the best immunoproteasome model achieved better results than the corresponding proteasome model when predicting the cleavage sites encompassed by an independent set consisting of HIV1-specific CD8 T cell epitopes (Figure 2). Taking into account all the above, the immunoproteasome model appears to be the most suitable to predict the C-terminus of CD8 T cell epitopes.

Our constitutive proteasome and immunoproteasome models produced different but overlapping fragmentation patters that mirror those observed experimentally [13]; 68% of the cleavage sites (*P1 *residues) and 36% of the fragments generated were identical (Figure 3B). However, the fragments yielded by the immunoproteasome and proteasome models were much smaller (2-3 residues) than those determined experimentally (7-9 residues) [13,47]. The smaller fragment sizes produced by our models may reproduce the clustering and overlapping of epitopes found in protein regions [48]. On the other hand, it is important to note that our models are not meant, and are not suitable, to predict proteolytic fragments, but to indicate whether the C-terminus of a peptide can result from the cleavage produced by the proteasome and/or the immunoproteasome. Proteasome fragmentation patterns (the size of fragments) may be better reproduced by methods trained on actual cleavage data such as that by Tenzer *et al *[18].

Using a test set of HIV1-specific CD8 T cell epitopes, we found that the predictive performance of our optimal proteasome and immunoproteasome cleavage models was comparable to that of NetChop [22]; a reference method to predict proteasome cleavage sites [24] that it was also developed from MHCI-restricted peptides. The immunoproteasome and proteasome cleavage models achieved *MCC *values of 0.20 and 0.19, respectively, while NetChop achieved an *MCC *= 0.18. It is worth nothing that these results were obtained under conditions that were optimal for NetChop. First, NetChop was trained on peptide fragments encompassing full length MHCI-restricted peptides [22], and here we have evaluated and compared the cleavage predictions over the entire epitope sequences. Note that we only used a portion of the MHCI-restricted peptides for training (6 residues). Second, the HIV1-specific CD8 T cell epitopes used for testing were not used for training our *N-gram *models but were likely included in the NetChop training dataset. It is also important to mention that NetChop has been described as an immunoproteasome cleavage prediction method, but in fact it was trained on a dataset consisting of both, MHCI-eluted ligands and CD8 T cell epitopes. As we have discussed here, CD8 T cell epitopes can be considered as generated by the immunoproteasome. However, it is more appropriated to consider MHCI-eluted peptides as generated by the constitutive proteasome because they are obtained from different type of cells but seldom from dendritic cells. In sum, we have dealt with the prediction of proteasome and immproteasome cleavage sites from MHC-restricted peptides in a manner that is consistent with the mechanism of antigen presentation and recognition, and achieved a notorious performance.

Prediction of proteasome and immunoproteasome cleavage sites using our models is available at http://imed.med.ucm.es/Tools/PCPS/. In addition, there are several other online servers to predict proteasome cleavage, which differ in the data and approach used for generating the models [16,22,49]. Nonetheless, the problem of identifying proteasome cleavage sites with high precision is still far from being solved. A simple manner to improve the prediction of proteasome cleavage sites could likely be achieved trough a meta-server that would arrive to a consensus prediction from the available proteasome cleavage predictors. Such a consensus approach has resulted successful in the also difficult task of predicting peptide binding to MHC class II molecules [50].

It has been reported that proteasome prediction models can improve T cell epitope identification when combined with MHCI-peptide binding predictions [18,22,37,51,52]. Likewise, our proteasome and immunoproteasome models, separately or together, also served to improve CD8 T cell epitope discrimination when combined with MHCI-binding predictions (Figure 5). The improvements, judged by increases in *AUC*, could appear minor but were statistically significant (Figure 5), and were linked to a large reduction of the number of false positives detected (up to 70%). Therefore, combining proteasome cleavage and MHCI-peptide binding predictions would serve to decrease the experimental toll involved in epitope identification; there will be less peptides to be tested. The proteasome cleavage model alone or juxtaposed with the immunoproteasome model resulted in a significant loss of true positives (up to 20%). Therefore, the proteasome cleavage model will be more useful on large-scale epitope identification scenarios (e.g. predicting CD8 T cell epitopes from a large number of antigens). Finally, combining cleavage predictions by both proteasomes, constitutive and immunoproteasome, with MHCI-binding predictions ought to help defining protective CD8 T cell epitopes. Overall, these results call for the integration of our proteasome models with others taking into account TAP transport and MHC binding, as already pioneered by other authors [18,22,37,51,52].

## Conclussion

We have derived *N-gram *models specific for the proteasome and the immunoproteasome that are consistent with the known biology of antigen presentation. The proteasome models were built upon MHCI-eluted peptides whereas the immunoproteasome models were built upon CD8 T cell epitopes. The *N-gram *models that exhibited the best performance were trained on 12-residue peptides, 6 residues at each side of the cleavage site, defined by the C-terminus of MHCI-restricted peptides and the most proximal C-terminal flanking residue. Finally, we have shown that combining cleavage predictions by the proteasome and immunoproteasome models with MHCI-binding predictions improves CD8 T cell epitope prediction. Cleavage predictions using our *N-gram *models are available for free public use at the PCPS site http://imed.med.ucm.es/Tools/PCPS/.

## Abbreviations used

MHC: I molecules, major histocompatibility class I molecules; N-terminus: amino-terminus; C-terminus: carboxy-terminus.

## Authors' contributions

CMDR did the work and wrote paper. EML interpreted results and wrote paper. PAR designed the work, interpreted results and rendered the final paper. All authors read and approved the final manuscript.

## Supplementary Material

Adittional file 1 **MHCI allele distribution in peptide datasets**. The figure depicts the percentage of peptides restricted by 7 commonly expressed human MHCI alleles (A*0201, A*0301, A*1101, A*2402, B*0702, B*0801, B*2705) in the three datasets used in this study. Click here for file
