# Supplementary material for: Computational analysis and modeling of cleavage by the immunoproteasome and the constitutive proteasome
Source: BMC Bioinformatics. 2010 Sep 23;11:479. doi: 10.1186/1471-2105-11-479 (PMC2955702; doi:10.1186/1471-2105-11-479)
Supplement: Adittional file 1 — MHCI allele distribution in peptide datasets. The figure depicts the percentage of peptides restricted by 7 commonly expressed human MHCI alleles (A*0201, A*0301, A*1101, A*2402, B*0702, B*0801, B*2705) in the three datasets used in this study. [file 1471-2105-11-479-S1.PDF]

Peptides (%)

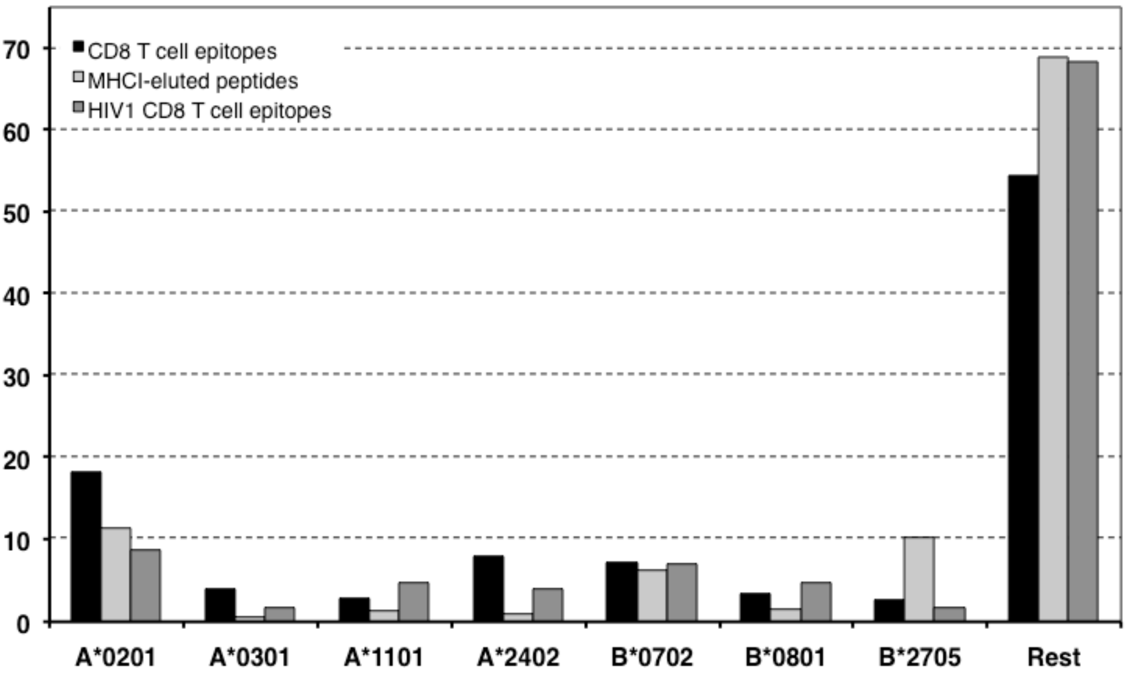

Additional file 1. MHCI allele distribution in peptide datasets. The restricting elements (MHCI alleles and antigens) included in the three datasets are: 80 in the dataset of CD8 T cell epitopes from all sources but HIV1 (first dataset), 71 in the dataset of MHCI-eluted peptides (second dataset) and 48 in the dataset of HIV1-specific CD8 T cell epitopes (third dataset or independent dataset). In average, not a single MHCI allele and/or antigen restricts more than 11% of the total peptides in any of the datasets, with the exception A*0201, which restricts 18% of the epitopes (in the first dataset). The figure depicts the distribution in percentage of peptides restricted by seven commonly expressed MHCI alleles (A*0201, A*0301, A*1101, A*2402, B*0702, B*0801, B*2705) in the three datasets: CD8 T cell epitopes from all sources but HIV1 (*black bars*), MHCI-eluted peptides (*light grey bars*) and HIV1-specific CD8 T cell epitopes (*dark grey bars*). The last column (*Rest*) accounts for all the remaining peptides restricted by all MHCI molecules but those specified in the graph.
